# Reduced Consolidation, Reinstatement, and Renewal of Conditioned Fear Memory by Repetitive Treatment of Radix Polygalae in Mice

**DOI:** 10.3389/fpsyt.2017.00097

**Published:** 2017-05-31

**Authors:** Jung-Won Shin, Hyunwoo Park, Yoonju Cho, Suck Lee, Jiwon Yoon, Sungho Maeng

**Affiliations:** ^1^Department of Gerontology, Graduate School of East-West Medical Science, Kyung Hee University, Yongin, South Korea; ^2^Center for Nutraceutical and Pharmaceutical Materials, Myongji University, Yongin, South Korea; ^3^Department of East-West Medicine, Graduate School of East-West Medical Science, Kyung Hee University, Yongin, South Korea; ^4^East-West Medical Research Institute, Kyung Hee University, Seoul, South Korea

**Keywords:** posttraumatic stress disorder, conditioned fear, Radix Polygalae, reinstatement, renewal, extinction

## Abstract

The therapeutic goal for the treatment of posttraumatic stress disorder (PTSD) is to promote extinction and to prevent the relapse of fearful memories. Research has identified pharmacological treatments that may regulate the formation and extinction of fear memories, but not many reagents that block the relapse of extinguished fear are known. Radix Polygalae (RP) is an Asian herb used for sedation, and its ingredients have anxiolytic and antidepressant properties. As various neurological effects have been identified, we tested whether RP affects the relapse of fear. Freezing in response to a conditioned context and cues was used to measure the effects of RP in mice. In cohort 1 (*n* = 30), consolidation, extinction, and reinstatement were tested during the course of 18 days of treatment. In cohort 2 (*n* = 30), consolidation, extinction, and renewal were tested during 10 days of treatment. The consolidation, extinction, reinstatement, and possibly the renewal of context-induced freezing were inhibited due to the administration of RP in animal subjects. However, the effects of RP on the freezing responses of subjects elicited by conditioned auditory cues were less obvious. Because it effectively suppresses the consolidation of fear memories, RP may be used for primary and secondary prevention of symptoms in PTSD patients. Additionally, because it effectively suppresses the reinstatement and renewal of fear memories, RP may be applied for the prevention of fear relapse in PTSD patients who have undergone exposure therapy.

## Introduction

Fear is an emotion that makes us alert to potential threats. In contrast to preborn innate fear, acquired fear is characterized by associative learning about pertinent stimuli during a fearful threat (1). Once acquired, sensory cues can elicit fear, despite the absence of practical threats. Fear conditioning is a well-recognized research tool into fear memory (2). While electric foot shocks (EFSs), an example of an unconditioned stimulus (US) are inflicted, environmental context and sounds, the conditioned stimulus (CS), become conditioned cues that induce fear responses. Using these cues, consolidation, extinction, reinstatement, and renewal of fear can be tested (3).

Consolidation is the long-term storage of fear, which requires protein synthesis (4). Once consolidated, confrontations with the CS will elicit anticipatory fear. Meanwhile, in the absence of the US, repeated presentations of the CS lead to a gradual decline of anticipatory fear responses (phenomenon known as extinction). However, extinguished fear return with the passage of time (spontaneous recovery), following an aversive event (reinstatement) and when the CS is presented in a different context (renewal) (5). This return of fear is evidence that extinction is not an erasure but rather indicates that a subject has relearned the CS-US association, thereby inhibiting the prior fear response (6). Clinically speaking, the return of fear in subjects following exposure-based therapies is a common problem, which contributes to relapse in anxiety disorders. Thus, research on fear relapse regulation is required.

Radix Polygalae (RP) is an herb used in traditional medicine. Recently, neuroprotective, cognitive improvement, antidepressant like, and anxiolytic effects have been revealed from RP and its components (7, 8). It has also been suggested that RP can suppress the acquisition and consolidation of fear memories in a mouse model of posttraumatic stress disorder (PTSD) (in press). Onjisaponins, 3-6′-disinapoyl sucrose (DISS), and 3,4,5-trimethoxycinnamic acid (TMCA) are the main components of RP extract. Among these, tenuifolin, which is the chemical backbone of onjisaponins, increased the synaptic level of dopamine and norepinephrine in the hippocampus (9). Also, antidepressant-like effect of DISS was related to the regulation of hypothalamus–pituitary–adrenal (HPA) axis and monoamine oxidase-B activity (10). Especially, TMCA modulated the activity of locus coeruleus by suppressing norepinephrine release (11). According to these effects on the hippocampus, HPA axis, and locus coeruleus, modulation of stress reactivity and fear response was suggested.

The current research tests the effects of RP on consolidation, extinction, reinstatement, and renewal using a model based on fear conditioning. In the first cohort (*n* = 30), fear consolidation, extinction, and reinstatement were tested, and in the second cohort (*n* = 30), consolidation, extinction, and renewal were tested. We demonstrated fear regulatory effects of RP through these experiments.

## Materials and Methods

### Animals

Seven-week-old male C57Bl/6 mice [Central Laboratory Animals Inc., Korea (*n* = 60)] were housed in a temperature-controlled and humidity-controlled environment under 12-h light–dark cycles with free access to food and water. Protocols were approved (KHMC-IACUC: 12-009) and thus conducted in accordance with the NIH Guide for the Care and Use of Laboratory Animals. In cohort 1, each 10 mice were randomly assigned to control (DW), RP0.1 (RP 0.1 mg/kg), and RP1 (RP 1 mg/kg) groups. In cohort 2, each 10 mice were randomly assigned to control (DW), RP1 (RP 1 mg/kg), and RP10 (RP 10 mg/kg) groups.

### Preparation of RP Extract

Radix Polygalae was purchased from Kyung Hee Hanyak (Korea). A voucher specimen was deposited. Dried RP was boiled at 80°C in 70% ethanol for 1 h, and the process was repeated for 40 min. The combined filtrate was evaporated on a rotary evaporator under reduced pressure and freeze-dried to yield 28% (w/w) of the original product. For experimental use, crude extracts were dissolved in DW at a concentration of 10 µg/mL (w/v).

### Fear Conditioning, Extinction, and Reinstatement

**Figure 1:**
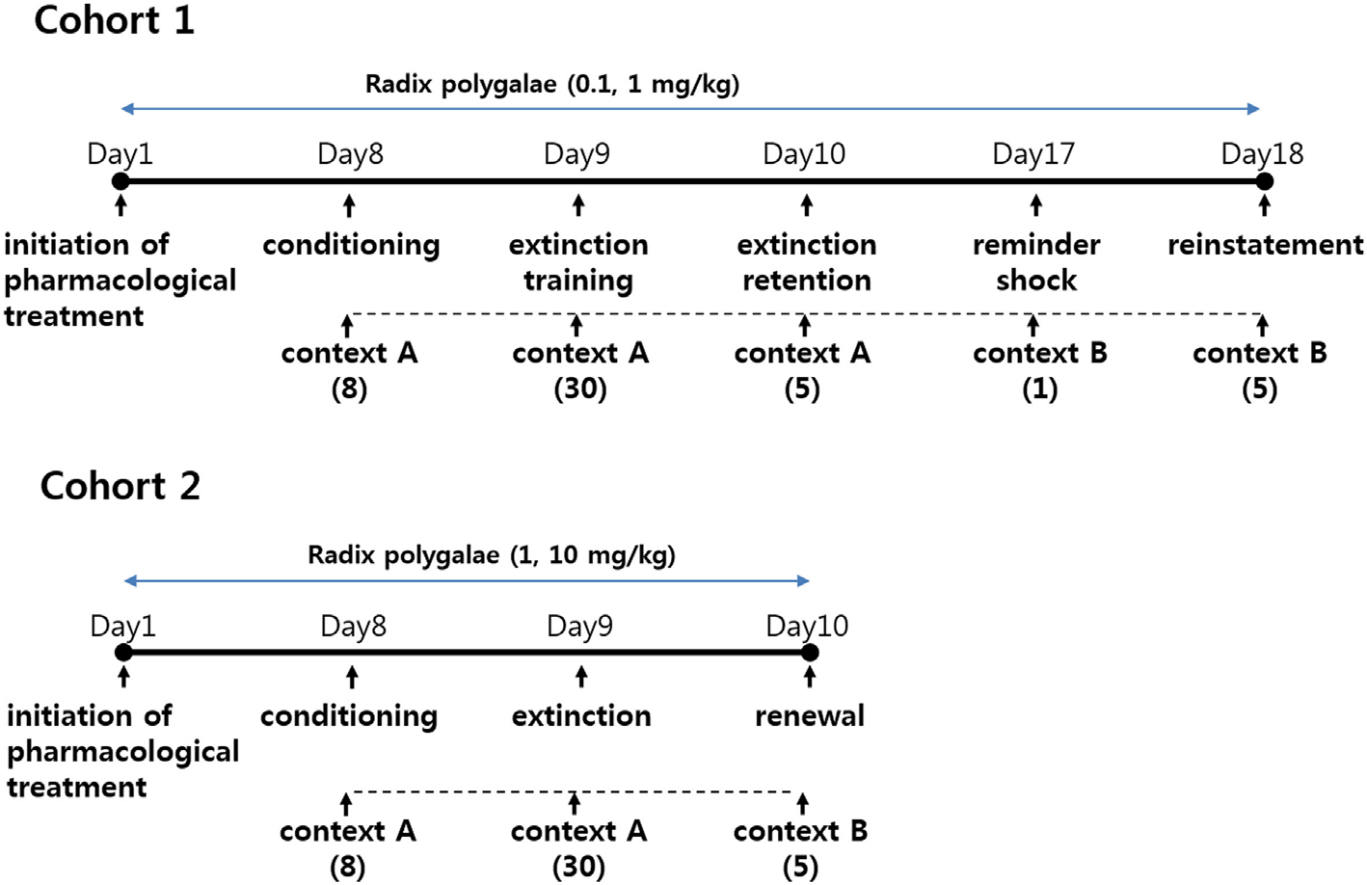
Schematic of experimental schedules.

In cohort 1, freezing responses to context and auditory cues were recorded during and after the conditioning of subjects to EFS. RP (0.1 and 1 mg/kg) or vehicle (DW) was given orally (gastric lavage) for 18 days. As depicted in Figure 1, all behavioral procedures were performed in a conditioning chamber (context A), beginning 8 days after the initiation of pharmacological treatment. The conditioned response of consolidation, extinction, and extinction retention was measured during the course of 3 consecutive days. A week later, a reminder shock was delivered, and the level of reinstatement was measured 24 h later.

For the acquisition of fear, mice were placed in the chamber and left undisturbed for 2 min. Subsequently, sessions comprising CS-US (30 s) and intertrial intervals (ITI; 30 s) were repeated eight times. For CS, white noise (80 dB) was used. Twenty-eight seconds after the initiation of noise, an EFS (0.45 mA), which lasted for 2 s, was coterminated with the noise. Freezing behavior was measured during the initial 2 min in the context (baseline) and during CS-US (cued acquisition). For extinction, mice were placed back in the chamber 24 h later, and left undisturbed for 2 min. Subsequently, sessions of CS (30 s) that were not paired with US and followed by ITI (30 s) were repeated 30 times. Freezing was measured during the initial 2 min in the context (contextual consolidation) and during periodical CS (cued consolidation and extinction). For the measurement of extinction retention, mice were placed in the chamber 24 h later and left undisturbed for 2 min. Subsequently, sessions of CS (30 s) followed by ITI (30 s) were repeated five times. Freezing behaviors were measured during the initial 2 min in the context (contextual extinction retention) and during periodical CS (cued extinction retention). After a week of being left undisturbed in their home cage, the mice were brought back to the chamber, and a single instance of EFS (0.65 mA, 1 s) was administered. Next day, for the measurement of reinstatement, mice were placed in the chamber and were left undisturbed for 2 min. Subsequently, CS (30 s) and ITI (30 s) sessions were repeated five times. Freezing behaviors were measured during the initial undisturbed 2 min (contextual reinstatement) and during periodical CS (cued reinstatement). Freezing was defined as a complete lack of activity, with the exception of the respiratory movements of the rib cage.

### Fear Conditioning, Extinction, and Renewal

In cohort 2, conditioned freezing of consolidation, extinction, and renewal in context and auditory cues was measured. RP (doses of 1 and 10 mg/kg p.o.) or the vehicle (DW) was administered by gastric lavage for 10 days. As depicted in Figure 1, conditioning and extinction training were performed in the conditioning chamber (context A), but renewal was tested in a triangular-shaped chamber (context B). Behavioral procedures for fear conditioning began on the eighth day of treatment with RP.

For the acquisition, mice were placed in the conditioning chamber and left undisturbed for 2 min, then sessions of 30 s CS-US and 30 s ITI were repeated eight times. Freezing responses were measured during the initial 2 min in the context (contextual baseline) and during the CS-US (cued acquisition). For extinction, mice were placed in the chamber 24 h later and left undisturbed for 2 min. Subsequently, sessions of CS and ITI (both 30 s) were repeated 30 times. The mice were left in the chamber for an additional 2 min. Freezing was measured during the initial 2 min (contextual consolidation), during the 30 sessions of CS (cued consolidation and extinction) and during the additional 2 min in the context (contextual extinction). For renewal, mice were placed 24 h later in context B and left undisturbed for 2 min. Subsequently, sessions of 30 s CS and 30 s ITI were repeated five times, followed by an additional 2 min in the context. Freezing behavior was measured during the CS (cued renewal) and during the additional context exposure (contextual renewal).

### Statistics

Before statistical analysis, if the assumption of normality in distribution, checked by Shapiro–Wilk test, was violated, data were transformed into logarithmic scale to correct the skewness of distribution. If the assumption of homogeneity failed by Levene’s test for equality, Tamhane’s T2 test for *post hoc* analysis. If assumption of sphericity failed by Mauchly’s test, Greenhouse-Geisser effect was used for comparison between data. Treatment effect was compared as between-subject variables and procedures of fear regulation were compared as within-subject variables. The alpha value was set at *p* < 0.05. GPower 3.1 was used for power analysis with power (1-β) set at 0.8.

## Results

### RP Inhibits Reinstatement of Conditioned Fear

**Figure 2:**
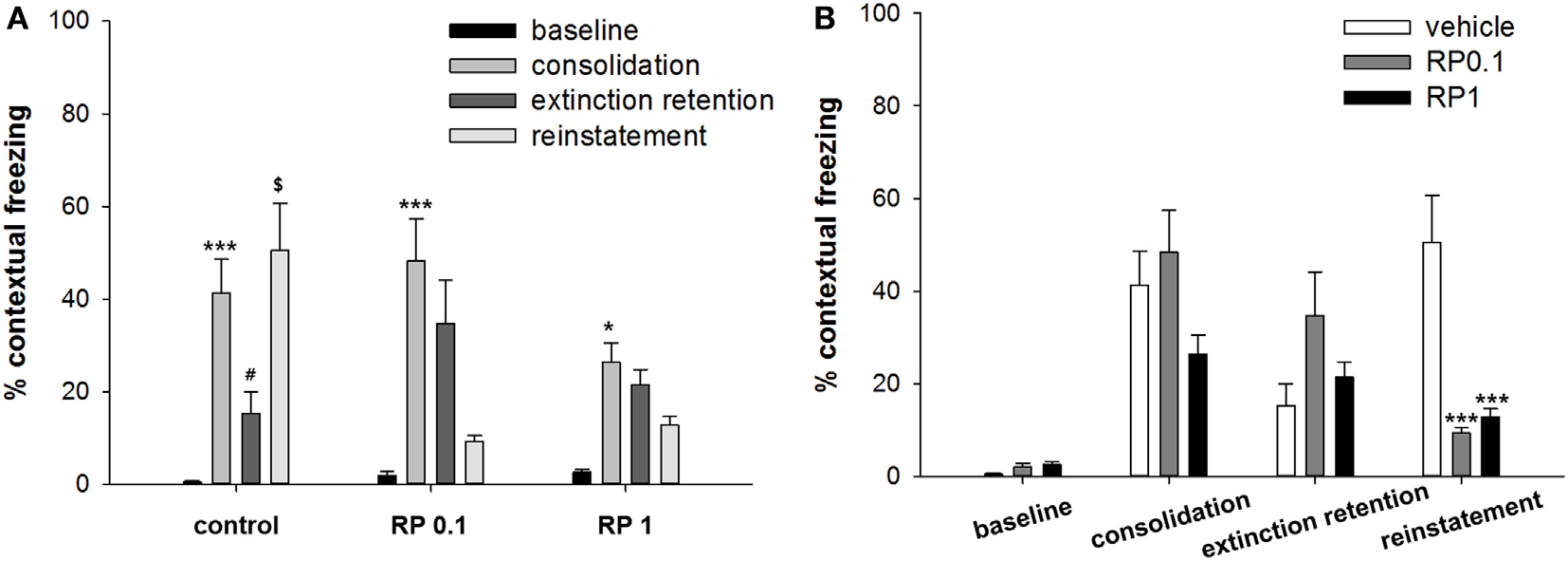
The effect of Radix Polygalae (RP) on contextual fear memory consolidation, extinction, and reinstatement. Repeat measure analysis of variance (ANOVA) showed within-group (procedure) effect [*F*(1.2, 33.1) = 87.0, *p* < 0.001, η^2^ = 0.763], between-group (treatment) effect [*F*(2,27) = 4.3, *p* = 0.023, η^2^ = 0.244], and within–between group interaction [*F*(2.5, 33.1) = 4.95, *p* = 0.009, η^2^ = 0.268]. **(A)** Within-group comparison of % freezing. In control group, fear memory was acquired (basal vs. consolidation, *p* < 0.001), extinct (consolidation vs. extinction, *p* = 0.024), and reinstated (extinction vs. reinstatement, *p* = 0.011). In RP0.1 group, fear memory was acquired (*p* < 0.001) but not extinct (*p* = 0.66) nor reinstated (*p* = 0.11). In RP1 group, fear memory was acquired (*p* = 0.011) but not extinct (*p* = 0.99) nor reinstated (*p* = 0.9). *p < 0.05, ***p < 0.001 vs. baseline. ^#^*p* < 0.05 vs. consolidation. ^$^*p* < 0.05 vs. extinction. **(B)** Between-group comparison of% freezing. There were no statistical difference among groups in the baseline, consolidated, and extinct level of contextual freezing, but freezing by the reinstatement of fear memory was lower in both RP0.1 (control vs. RP0.1, *p* < 0.001) and RP1 (control vs. RP1, *p* < 0.001). ***p < 0.001 vs. control. Data represent mean ± SEM. control: DW; RP0.1: Radix Polygalae 0.1 mg/kg p.o. 18 days; RP1: Radix Polygalae 1 mg/kg p.o. 18 days.

**Figure 3:**
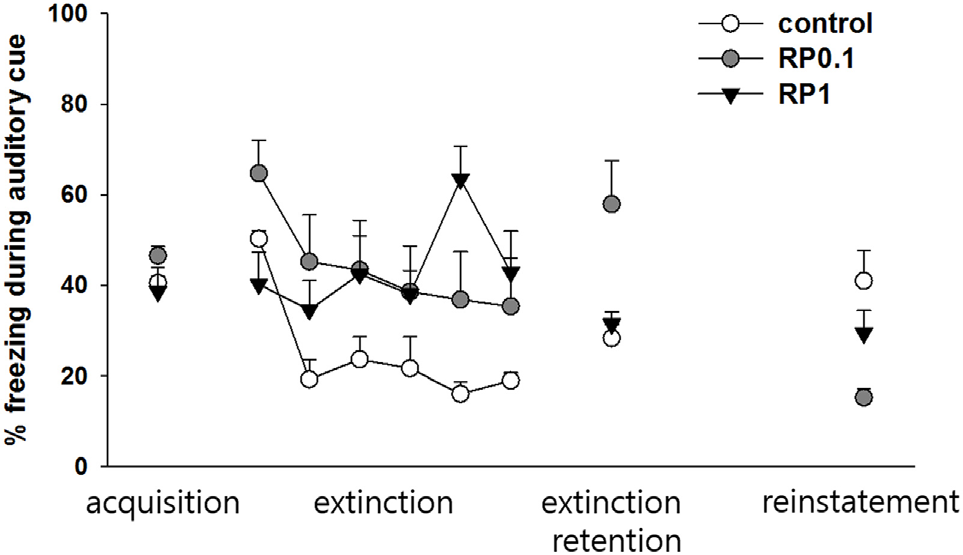
The effect of Radix Polygalae (RP) on cued fear memory acquisition, extinction, extinction retention, and reinstatement. The average % freezing of eight sessions (acquisition) or five sessions (extinction, extinction retention, and reinstatement) are plotted. Repeat measure analysis of variance (ANOVA) showed within-group (procedure) effect [*F*(2.9, 79.4) = 9.1, *p* < 0.001, η^2^ = 0.253] and within–between group interaction [*F*(5.9, 79.4) = 6.8, *p* < 0.001, η^2^ = 0.336] but no between-group (treatment) effect [*F*(2,27) = 2.12, *p* = 0.13, η^2^ = 0.138]. Within the control group, freezing decrease by extinction training (*p* < 0.001) but extinction retention and reinstatement were non-significant. Within the RP0.1 group, freezing decreased by extinction training (*p* < 0.001), non-significant increase in extinction retention measure (*p* = 0.086) and decrease by reinstatement (*p* = 0.001). Within the RP1 group, no change in either extinction, extinction retention, or reinstatement were detected. Data represent mean ± SEM. control: DW; RP0.1: Radix Polygalae 0.1 mg/kg p.o. 18 days; RP1: Radix Polygalae 1 mg/kg p.o. 18 days.

In the first cohort of mice, conditioned freezing responses of fear consolidation, extinction, extinction retention, and reinstatement were measured during the period of exposure to context and cues (Figures 2 and 3). Repeated measure analysis of variance (ANOVA) of the contextual freezing showed significant within-group (before-after) effects (*p* < 0.001, η^2^ = 0.763), between-group (experimental group) effects (*p* = 0.023, η^2^ = 0.244), and within–between group interaction (*p* = 0.009, η^2^ = 0.268) (Figure 2). Power analysis revealed that in order for an effect of these size to be detected (80% chance) as significant at the 5% level, a sample of 6 was required for the within-group and between-group effect and a sample size of 9 for the within–between group interaction. Within the vehicle treated control subjects, fear memories were consolidated (baseline versus consolidation, *p* < 0.001), extinguished (consolidation versus extinction retention, *p* = 0.024), and reinstated (extinction retention versus reinstatement, *p* = 0.011). Within both the RP0.1 and RP1 groups, fear memories were consolidated (*p* < 0.001, *p* = 0.011, respectively), but were neither extinguished nor reinstated (Figure 2A). Between experimental groups, there were no differences in the levels of baseline, consolidation and extinction retention, but the reinstatement of fear was lower in both the RP0.1 (*p* < 0.001) and RP1 (*p* < 0.001) groups (Figure 2B).

Repeated measures ANOVA of the CS-induced freezing showed significant within-group effects (*p* < 0.001, η^2^ = 0.253) and within–between group interactions (*p* < 0.001, η^2^ = 0.336), but non-significant between-group effects (*p* = 0.13, η^2^ = 0.138) (Figure 3). Power analysis revealed that in order for an effect of these size to be detected (80% chance) as significant at the 5% level, a sample of 6 was required for the within-group, 9 for the between-group, and 6 for the within–between group interaction. Within the control group, fear memories were shown to be extinct (average freezing during the first to fifth sessions versus average freezing during the 26–30th sessions of extinction training, *p* < 0.001), but extinction retention (average freezing during the 26th to 30th sessions of extinction training versus average freezing during the five sessions of extinction retention) and reinstatement (average freezing during the five sessions of extinction retention versus average freezing during the five sessions of reinstatement) were not significant. Within the RP 0.1 group, fear memories were shown to be extinct (*p* < 0.001), but extinction retention (*p* = 0.086) was not significant. In addition, a reminder shock did not induce reinstatement in this group. Within the RP1 group, no significant change in freezing was detected for consolidation, extinction, or reinstatement. Based on these results, RP inhibits the reinstatement of conditioned fear and also inhibits the consolidation of fear memories with the administration of 1 mg/kg in the context but has no effect during periods of cued exposure among animal subjects.

### RP Inhibits the Consolidation or Renewal of Contextual Conditioned Fear

**Figure 4:**
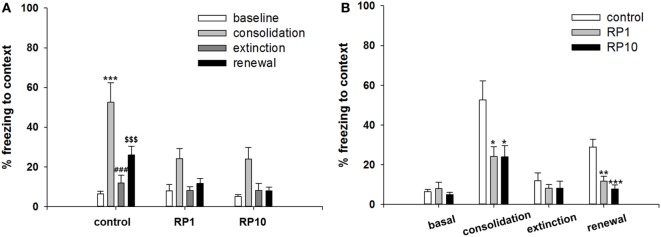
The effect of Radix Polygalae (RP) on contextual fear memory consolidation, extinction and renewal. Repeat measure analysis of variance (ANOVA) showed within-group (procedure) effect [*F*(3, 81) = 27.5, *p* < 0.001, η^2^ = 0.505], between-group (treatment) effect [*F*(2,27) = 5.3, *p* = 0.011, η^2^ = 0.282] but no within–between interaction [*F*(6, 81) = 1.7, *p* = 0.14, η^2^ = 0.110]. **(A)** Within-group comparison of % freezing. In control group, fear memory was acquired (baseline vs. consolidation, *p* < 0.001), extinct (consolidation vs. extinction, *p* < 0.001), and renewed (extinction vs. renewal, *p* < 0.001). In RP1 and RP10 group, fear acquisition, extinction and renewal were all non-significant. ***p < 0.001 vs. baseline. ^###^*p* < 0.001 vs. consolidation. ^$$$^*p* < 0.001 vs. extinction. **(B)** Between-group comparison of % freezing. Consolidated fear were lower in RP1 (*p* = 0.027) and RP10 (*p* = 0.026) compared by controls. Renewed fear were also lower in RP1 (*p* = 0.001) and RP10 (*p* < 0.001) compared by control. There were no difference in the baseline and extinct level of fear response. *p < 0.05, **p < 0.01, and ***p < 0.001 vs. control. Data represent mean ± SEM. control: DW; RP1: Radix Polygalae 1 mg/kg p.o. 10 days; RP10: Radix Polygalae 10 mg/kg p.o. 10 days.

**Figure 5:**
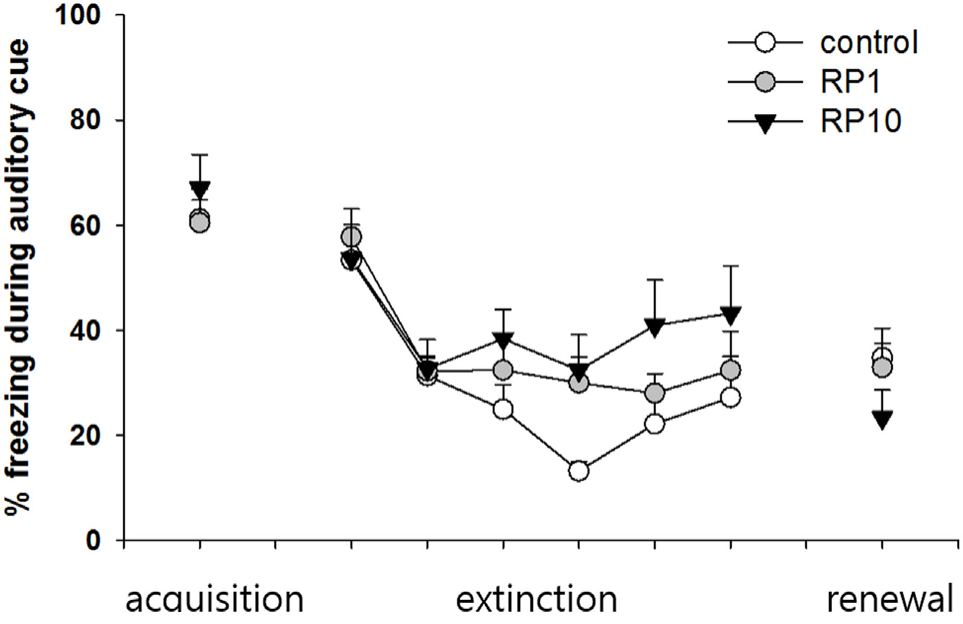
The effect of Radix Polygalae (RP) on cued fear memory acquisition, extinction and renewal. The average % freezing of eight sessions (acquisition) or five sessions (extinction and renewal) are plotted. Repeat measure analysis of variance (ANOVA) showed within-group (procedure) effect [*F*(3.5, 94.3) = 17.3, *p* < 0.001, η^2^ = 0.391] but non-significant between-group (treatment) effect [*F*(2,27) = 0.73, *p* = 0.49, η^2^ = 0.052] and within–between interaction [*F*(7.0, 94.3) = 2.0, *p* = 0.06, η^2^ = 0.13]. In the control group, fear reduced during the extinction training (first extinction training block vs. sixth extinction training block; *p* = 0.048), but fear did not increase during the renewal procedure. In both RP1 and RP10, fear extinction and fear renewal were not significant (fear extinction in RP1; *p* = 0.062). Data represent mean ± SEM. control: DW; RP1: Radix Polygalae 1 mg/kg p.o. 10 days; RP10: Radix Polygalae 10 mg/kg p.o. 10 days.

In the second cohort of mice, conditioned freezing responses of fear consolidation, extinction, and renewal were measured during the period of exposure to context and cues (Figures 4 and 5). Repeated measure ANOVA of the contextual freezing showed significant within-group effects (*p* < 0.001, η^2^ = 0.505), between-group effects (*p* = 0.011, η^2^ = 0.282), but not within–between group interaction (*p* = 0.14, η^2^ = 0.11) (Figure 4). Power analysis revealed that in order for an effect of these size to be detected (80% chance) as significant at the 5% level, a sample size of 6 was required for the within-group and between-group, and 18 for the within–between group interaction.

Within the control group, fear memories were consolidated (freezing in context A before electric shock conditioning versus freezing in context A before extinction training, *p* < 0.001), extinguished (freezing in context A before versus freezing after extinction training, *p* < 0.001), and renewed (freezing in context A after extinction training versus freezing in context B after CS sessions, *p* < 0.001). Within the RP1 and RP10 groups, freezing behaviors did not show significant changes associated with consolidation, extinction, and renewal (Figure 4A). Between experimental groups, consolidated fear levels were lower in both the RP 1 mg/kg (*p* = 0.027) and RP 10 mg/kg (*p* = 0.026) groups. In addition, renewed fear levels were lower in both RP1 (*p* = 0.002) and RP10 (*p* < 0.001) groups (Figure 4B).

Repeated measures ANOVA of the CS-induced freezing showed significant within-group effects (*p* < 0.001, η^2^ = 0.391) but showed neither between-group effects (*p* = 0.49, η^2^ = 0.052) nor within–between interactions (*p* = 0.06, η^2^ = 0.13) (Figure 5). Power analysis revealed that in order for an effect of these sizes to be detected (80% chance) as significant at the 5% level, a sample of 6 was required for the within-group, 15 for between-group and within–between group interaction.

Within the control group, fear was shown to be extinct (average freezing during the first to fifth sessions versus the twenty-sixth to thirtieth sessions of extinction training; *p* = 0.048), but was not renewed (average freezing during the 26–30th sessions of extinction training versus freezing during CS in context B). Within both the RP1 and RP10 groups, there were no significant changes in freezing to indicate extinction or renewal. Based on these results, RP reduces the consolidation or the renewal and consolidation of fear memories in a conditioned context, but not in cued fear memories in animal subjects.

## Discussion

Facilitated extinction of fear memories and maintenance of the extinct state are important therapeutic goals in treatment of anxiety disorders (12). Here, we report that RP inhibits the consolidation, reinstatement and renewal of conditioned fear memories.

Extinction learning and maintenance of extinct states determine the outcome of exposure therapy in subjects with PTSD (13). In contrast to the acquisition of fear, extinction is slowly acquired and is related to the amount of extinction training (14). Thus, accumulated extinction trials with short ITIs may weaken the level of extinction (15). Pharmacologically speaking, endocannabinoids are considered important in facilitating extinction. Subjects with PTSD have been shown to exhibit dysregulation of the endocannabinoid system (16). Behavioral and endocrine changes reflecting PTSD symptoms in animal models have demonstrated prevention by a cannabinoid 1 receptor agonist or genetic enhancement of endocannabinoids (17). d-Cycloserine has been shown to strengthen levels of extinction and to reduce the number of therapy sessions in subjects with PTSD and social anxiety (18). Additionally, compounds including fibroblast growth factor, methylene blue, and yohimbine may be useful for accelerating or strengthening fear extinction in subjects (19). Recently, factors associated with the relapse of extinguished fear have been reported. Pharmacological reagents, such as l-dopa, scopolamine, and agomelatine, as well as trophic factors such as brain-derived neurotrophic factors and fibroblast growth factor-2, have been mentioned for the effective prevention of the relapse of fear (19). Interestingly, chronic administration of fluoxetine has been shown to prevent stress-induced reinstatement of fear (20).

As a traditional psychological adaptogen, RP reduced scopolamine-induced cognitive impairments (21) and its ingredients, DISS has shown antidepressant-like effects, and TMCA has shown antistress effects (10, 11). In addition, we described how RP exerts rapid-onset antidepressant-like effects (8) and reduces fear acquisition in the animal model of single prolonged stress (in press). According to the results of the current research, RP inhibits fear reinstatement. In the first cohort, animals in the control group displayed freezing patterns indicating contextual fear consolidation, extinction and reinstatement. In contrast, animals treated with RP did not display freezing associated with fear reinstatement. Based on Vervliet et al. (13), we investigated other possible explanations of this reinstatement blockade. First, if the level of consolidated fear was less, then the level of reinstated fear would also be less. In the findings herein, this is not probable because consolidation reached a significant level in all groups (based on within-group comparisons), which are not distinct among treatment groups (based on between-group comparisons). Second, if fear is insufficiently extinct, then residual fear may be misinterpreted as reinstatement. This also is not a probable explanation, because freezing after reinstatement was shown to be further reduced compared to freezing after extinction training. However, RP suppressed the retention of extinction memories. RP can seemingly suppress all kinds of fear associated with a context, which emerged in the analysis herein as lesser levels of consolidation, extinction, and reinstatement. In contrast, RP did not affect the conditioned responses to auditory cues. Within the control group, fear was acquired and extinguished, but no reinstatement was observed. In groups treated with RP, fear was acquired, but was not extinguished and was not reinstated. Although the between-group comparisons reveal no difference among groups, mice treated with RP showed lower levels of extinction and fewer freezing behaviors during the measures of reinstatement. This may indicate that cues related to fear memories are not affected by RP, or that a higher dose of RP is required to be meaningful. Other types of fear-related behaviors, such as rattling, were more frequently observed during the presentation of auditory cues, which may have confounded the results by reducing the freezing time (22).

According to the results from the second cohort, RP inhibited consolidation and possibly inhibited the renewal of contextual fear. Control animals displayed consolidation, extinction, and renewal in response to the conditioned context, but in the animals treated with RP, no measure of these behaviors was significant according to the within-group comparison. The between-group comparison, however, showed a significantly lower degree of extinction and renewal in the animals treated with RP. This may indicate that RP suppresses consolidation or suppresses both consolidation and renewal of contextual fear. Similar to the findings of cohort 1, auditory cue-induced freezing was not affected. In addition, all changes in the freezing behavior associated with fear extinction and renewal were insignificant.

According to the results from both cohorts, consolidation was suppressed by 10 mg/kg but not by 0.1 mg/kg of RP. The results of 1 mg/kg of RP treatment over the course of 18 days showed significant suppression (cohort 1, *p* = 0.011), but the results of treatment with this dosage for 10 days showed non-significant suppression of consolidation (cohort 2, *p* = 0.062). This difference may be a consequence of the duration of treatment. Extinction was suppressed in all occasions, both when the fear was consolidated (cohort 1) and when it was not (cohort 2). In addition, fear relapse was suppressed in comparison to control animals unrelated to the extent of consolidation and extinction.

In accordance with the traditional use of RP as an adaptogen, our results show that RP can be used to treat patients with PTSD associated with symptoms of excessive fear memory. In order to treat this disorder, either the consolidation or reconsolidation of fear should be suppressed or extinction should be facilitated. However, the method of inhibiting the consolidation has limited practical application because it is difficult to predict the fear inducing experience in advance. Also, several pharmacological attempts to promote the extinction of fear memory have yet to be effective with hazard of drug abuse (16). Therefore, recent studies have focused on finding drugs to suppress the recurrence of extinguished fear. Long-term exposure to fear-inducing signals may clear fear memory, but it can easily recur when the environment changes. This is a common problem in PTSD contributing to its difficulty to cure. As RP inhibited the recurrence of extinguished fear memory in animal models, RP may suppress the relapse of fear in PTSD patients undergone exposure therapy.

Among the known effects of RP, it is presumed that stress regulatory effects may be closely related to the inhibition of fear memory relapse. RP suppressed depression-like symptoms in chronic mild stress model mice and its saponin components showed antianxiety effects (7, 8). DISS has been reported to regulate the HPA axis and TMCA can regulate the activity of Locus coeruleus (10, 11). Polygalasaponin can also improve hippocampal-dependent learning and memory (23, 24). Probably fear memory consolidation and fear recurrence may depend on the amount of stress reaction of individual subjects. If RP can inhibit the stress reactivity when fear reaction was triggered, it may reduce the consolidation and recurrence of fear memory. More research of the molecular mechanisms in fear memory trace formation will provide better understanding about the pharmacologic mechanism of RP.

In summary, RP was found to (1) suppress contextual but not cue-induced fear, (2) suppress contextual fear consolidation in a manner that is dose dependent, (3) suppress contextual fear extinction, and (4) suppress the relapse of both reinstated and renewed fear in a conditioned context. These effects may be usefully implemented in the treatment of anxiety disorders associated with exaggerated fear. Suppression of fear consolidation may be a useful means for primary prevention in subjects at high risk for developing PTSD or for secondary prevention in subjects who have been previously exposed to traumatic stress. Suppression of fear relapse may be effective in PTSD patients who have completed exposure therapy.

## Ethics Statement

Protocols were approved by Kyung Hee Medical center—Institutional Animal Care and Use Committee (KHMC-IACUC: 12-009) and thus conducted in accordance with the NIH Guide for the Care and Use of Laboratory Animals.

## Author Contributions

J-WS and SL performed the experiments. HP and SM conceived and designed the experiments. YC and JY analyzed the data. SM wrote the article.

## Conflict of Interest Statement

The authors declare that the research was conducted in the absence of any commercial or financial relationships that could be construed as a potential conflict of interest.

## Abbreviations

ANOVA, analysis of variance; CS, conditioned stimulus; DISS, 3,6′-disinapoyl sucrose; EFS, electric foot shock; HPA, hypothalamus–pituitary–adrenal; ITI, intertrial interval; PTSD, posttraumatic stress disorder; RP, Radix Polygalae; TMCA, 3,4,5-trimethoxycinnamic acid; US, unconditioned stimulus.
